# Multi-Timescale Perceptual History Resolves Visual Ambiguity

**DOI:** 10.1371/journal.pone.0001497

**Published:** 2008-01-30

**Authors:** Jan W. Brascamp, Tomas H. J. Knapen, Ryota Kanai, André J. Noest, Raymond van Ee, Albert V. van den Berg

**Affiliations:** 1 Functional Neurobiology and Helmholtz Institute, Utrecht University, Utrecht, The Netherlands; 2 Department of Physics and Helmholtz Institute, Utrecht University, Utrecht, The Netherlands; 3 Biology Division, California Institute of Technology, Pasadena, California, United States of America; University of Minnesota, United States of America

## Abstract

When visual input is inconclusive, does previous experience aid the visual system in attaining an accurate perceptual interpretation? Prolonged viewing of a visually ambiguous stimulus causes perception to alternate between conflicting interpretations. When viewed intermittently, however, ambiguous stimuli tend to evoke the same percept on many consecutive presentations. This perceptual stabilization has been suggested to reflect persistence of the most recent percept throughout the blank that separates two presentations. Here we show that the memory trace that causes stabilization reflects not just the latest percept, but perception during a much longer period. That is, the choice between competing percepts at stimulus reappearance is determined by an elaborate history of prior perception. Specifically, we demonstrate a seconds-long influence of the latest percept, as well as a more persistent influence based on the relative proportion of dominance during a preceding period of at least one minute. In case short-term perceptual history and long-term perceptual history are opposed (because perception has recently switched after prolonged stabilization), the long-term influence recovers after the effect of the latest percept has worn off, indicating independence between time scales. We accommodate these results by adding two positive adaptation terms, one with a short time constant and one with a long time constant, to a standard model of perceptual switching.

## Introduction

The visual system adjusts its processing of current input on the basis of past experience. Such dynamic adjustment allows, for instance, faster responses to recurrent stimuli [1] and tuned weighting of visual cues depending on their previous validity [2]. A fundamental question for such adaptive systems is *how long* a history to incorporate in current processing.

**Figure 1:**
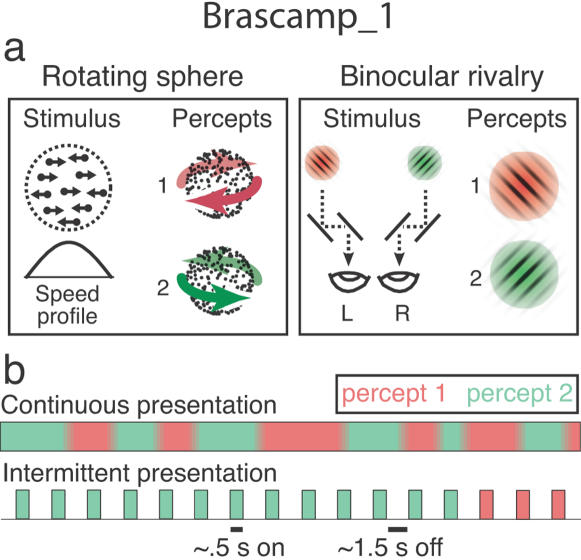
Stimuli and presentation sequences. a, Each of our stimuli has two distinct perceptual interpretations. Only one percept is experienced at any given moment. Left: An ambiguous rotating sphere is a two-dimensional projection of dots covering the surface of a transparent sphere that rotates around a central axis. Because no cue indicates which dots are in front, the rotation direction is ambiguous and subjects perceive either direction in turn, as indicated by the red and green arrows. Right: In binocular rivalry ambiguity arises because two incompatible images are projected into the two eyes (designated as ‘L’ and ‘R’). Subjects perceive the left eye's image or the right eye's image in turn. b, Top: Viewing an ambiguous stimulus continuously, observers experience random alternations between both percepts every few seconds. Bottom: Periodically removing the stimulus from view (here: on-time ∼0.5 s; off-time ∼1.5 s) causes perception to stabilize in one interpretation for sometimes minutes, with only incidental switches between alternative interpretations: Perceptual stabilization.

An opportunity to examine the role of history in vision within a controlled experimental setting is provided by ambiguous stimuli (Figure 1a). These images convey conflicting information to the eyes, causing perception to waver randomly between alternative interpretations, or *percepts* (Figure 1b, top). For instance, if a movie of a transparent revolving sphere with dots on its surface is stripped of all depth information, such as perspective and occlusion, it causes alternating perception of either possible rotation direction (Figure 1a, left). Alternatively, presenting incongruent images to the two eyes simultaneously causes alternating perception of either image in isolation (binocular rivalry; Figure 1a, right). Strikingly, prior experience can allow the state of perceptual indecision brought about by ambiguous stimuli to be overcome. That is, when observers are presented with an ambiguous stimulus they have viewed before, they often instantly perceive the same interpretation as they did on the prior encounter, even though the immediate visual input remains inconclusive. This can lead to prolonged periods of perceptual stabilization in case an ambiguous stimulus is periodically removed from view and the same percept keeps reappearing on consecutive presentations (Figure 1b, bottom) [3]–[9]. This salient expression of visual memory provides a convenient measure to study how traces of past perception interact with current input in shaping what we see.

Perceptual memory for ambiguous stimuli has a persistence of at least minutes, in the sense that even if an ambiguous stimulus does not reappear until several minutes after disappearing, the previous percept often still recurs [3]. Does this imply that a minute-scale perceptual history is incorporated in processing current visual stimuli? On the contrary, considering that the percept that is experienced (or *dominant*) at reappearance is generally simply the one that also dominated during the most recent encounter, one is tempted to conclude that only a single percept is stored, and that memory is ‘overwritten’ whenever perception changes (*switches*) to the alternative interpretation. Memory would then reach back no further than the moment of the latest switch. This is indeed implied by the common view that perceptual stabilization of ambiguous images reflects persistence after stimulus removal of the present state of perceptual organization [3]–[5], [7], [8]. It would appear that a system centered on persistence of the present dominance state could store only a single percept at a time.

Within the broader context of history dependence in vision this view is remarkable. If the goal of a visual memory system is to optimize processing based on past experience, storage of a single percept or event is of limited use. Processing would benefit from incorporating a more elaborate record of past events. Indeed, the literature does contain indications that persistence of the current perceptual state may be insufficient to explain history effects in ambiguous vision. For instance, in case an ambiguous stimulus is removed from view only shortly after a perceptual switch occurred (under about 2 s), the percept that dominated *before* the switch–not the most recent percept–often regains dominance at stimulus reappearance [3]. It is unknown what makes this particular manipulation exceptional, but the finding seems at odds with the idea that all memory is erased as soon as perception switches. Second, when an ambiguous stimulus is preceded by a sequence of stimuli that are similar but contain unequal evidence for either interpretation (biased stimuli), influences of several preceding stimuli on current perception can be measured [9]. Although the use of biased stimuli complicates the interpretation of the latter findings (see Discussion), they again suggest that traces of perceptual history may extend beyond a change in perception, raising the possibility of a memory system with greater functional merit.

**Figure 2:**
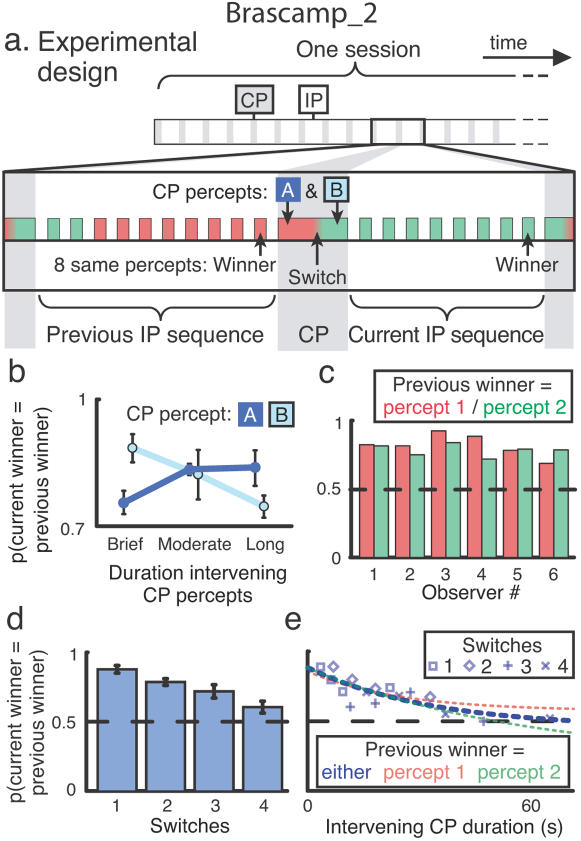
The effect of spontaneous perceptual switches on perceptual stabilization. a. Experimental design, Sequences of intermittent presentation (IP) were interleaved with periods of continuous presentation (CP). An intermittent presentation sequence ended when the same percept was reported on eight consecutive presentations. This percept was termed the sequence *winner*. In experiment one a continuous presentation period was terminated after a randomly varied delay (B) following the first perceptual switch. The duration (A) between the start of continuous presentation and this switch varied naturally. b. The probability that the current intermittent presentation sequence is won by the same percept as the previous intermittent presentation sequence rises with intervening duration A but falls with intervening duration B (both p<0.01; Spearman on individual subjects' data; n = 6; ρ = 0.68 for A and −0.56 for B). It remains above chance level throughout. Duration A varied continuously; the three data points were obtained by dividing the durations into three quantiles (average durations: 1.7, 4.1 and 11.6 s). c. The same probability plotted separately for cases where percept 1 (red) and percept 2 (green) won the previous intermittent presentation sequence. For each subject (x-axis) both bars reach above chance level, so the high correlation between the current winner and the previous winner does not reflect a systematic bias toward one percept. d. When allowing multiple perceptual switches (x-axis) during continuous viewing, the probability that the current winner equals the previous winner decreases as the number of intervening switches increases (p<0.01; Spearman on individual subjects' data; n = 7, ρ = −0.69). e. For one representative subject (others shown in Supporting Figure S2) the probability decreases gradually with increasing duration of the intervening continuous presentation period (x-axis), reaching chance after about a minute. This outcome is similar, whether one includes all data (blue curve and data points), or just cases where percept 1 (red curve) or percept 2 (green curve) won the previous intermittent presentation sequence. Error bars indicate standard errors.

We study how prior perception of an ambiguous stimulus influences how it is perceived at reappearance, specifically aiming at distinguishing persistence of the most recent percept from more intricate influences of past perception. We interleave episodes of intermittent viewing with episodes of continuous viewing (Figure 2a). During intermittent viewing perception stabilizes into one interpretation, whereas continuous viewing prompts spontaneous switches between percepts. The switches are essential, as they permit a dissociation between the most recent percept (following a switch) and preceding perception, enabling us to pit the effects of immediate and more remote perceptual history against each other.

## Results

### The influence of a spontaneous perceptual switch on stabilization

Two distinct ambiguous stimuli were used in our experiments (Figure 1). Results presented in the main text are for the ambiguous rotating sphere stimulus. Data from binocular rivalry are very similar (Supporting Text S1, Figures S1 and S3). The general layout of the sessions was the same for all experiments. It is illustrated in Figure 2a using experiment 1 as an example. Sessions consisted of blocks of intermittent presentation of an ambiguous stimulus, interleaved with periods of continuous presentation where perception was allowed to switch spontaneously. Subjects reported their perceptual state when a stimulus reappeared and whenever the percept switched. Transitions between intermittent presentation and continuous presentation were interactively initiated on the basis of observers' perceptual reports (Figure 2a, bottom). Intermittent presentation sequences proceeded until an observer reported the same percept on eight consecutive presentations, signaling robust stabilization. When this occurred, the stabilized percept was termed the *winner* of that intermittent presentation sequence, and continuous presentation was started. Continuous presentation periods, in turn, were terminated a fixed period after an observer reported a perceptual switch, and then intermittent presentation started again.

In the first experiment we allowed a single switch during continuous viewing. The delay (‘B’ in Figure 2a) between this switch and stimulus offset was drawn randomly from the values 0.5, 1.5 and 3 s. This mimicked the aforementioned situation in [3] where the effect of an incidental switch on stabilization was shown to depend on this delay. Perceptual switches naturally occur at random intervals, so the percept preceding the switch (‘A’ in Figure 2a) also had a variable duration. This allowed us to study how perception at stimulus reappearance depended on a well-controlled fraction of perceptual history; that is, on the duration of both perceptual episodes A and B.

As a main measure we will use the probability that the winner of a given intermittent presentation sequence equals the winner of the preceding intermittent presentation sequence. If it does not, the perceptual switch successfully disrupted perceptual stabilization; if it does, perception during this intermittent presentation sequence reverted to the winner of the previous intermittent presentation sequence in spite of the intervening switch to the opposite percept.

Figure 2b plots the subject-averaged probability that the winner of the current intermittent presentation sequence equals the previous winner, as a function of the intervening percept durations A and B; that is, the durations preceding and following the perceptual switch, respectively. The durations are categorized as ‘brief’, ‘moderate’ and ‘long’, as indicated on the x-axis. In case of percept B these three categories correspond simply to the three delay durations applied, whereas for percept A (whose duration varied in a continuous fashion) we divided the data up into three percentiles to form the three categories. The light curve shows that the current winner is less likely to equal the previous winner in case the period following the switch (percept B) is longer. In other words, the longer the final percept of the continuous presentation episode dominated before stimulus offset, the more likely it was to remain stabilized during the subsequent intermittent presentation sequence, replicating [3]. A new finding here is that the duration of the percept preceding the switch (percept A; dark curve) has the opposite effect. That is, the longer the previous winner remained dominant before the perceptual switch occurred, the more likely it was to regain dominance during the subsequent intermittent presentation sequence, in spite of the intervening switch. Note that the durations A and B were not correlated in this design, as the delay between the perceptual switch and stimulus offset was varied independently of the spontaneous percept duration preceding the switch. Both curves therefore reflect different, orthogonal, subdivisions of the same data set.

In isolation, the influence of the final percept duration (B) would be consistent with an explanation that during a blank the visual system retains its latest perceptual organization, provided this organization has had sufficient time to establish. The finding of a comparable influence of the preceding percept duration (A), however, argues against such an exceptional role for the final percept. Instead, it suggests that ambiguous figure memory is determined by a more global perceptual history. Specifically, Figure 2b leads to the tentative interpretation that the longer a percept has dominated in the past–be it during the final dominance period or earlier–the more readily it will regain dominance when the stimulus reappears.

Apart from the effects of durations A and B, Figure 2b also shows a general tendency for the winner of the current intermittent presentation sequence to equal the previous winner; that is, all points lie above 0.5. Figure 2c addresses whether this could be due to a systematic tendency for subjects to report one particular percept during intermittent presentation [10], regardless of history. For individual subjects this panel shows the probability that the current winner equals the previous one, both for the cases where percept 1 won the previous intermittent presentation sequence (red) and those where percept 2 did (green). Both the red and the green bars all reach beyond 0.5. This demonstrates that the current winner generally equaled the previous winner regardless of which percept it was, ruling out systematic bias as an explanation. As an extra measure we calculated each subject's individual bias, as the overall fraction of intermittent presentation sequences won by that subject's more predominant percept. This value was 0.57 on average, which is insufficient to explain the values in Figure 2b. It is striking to note that even the subject with the strongest systematic bias (0.69) still showed above-chance recurrence of the previous winner, even in case this winner was the weaker of the two percepts (observer 4 in Figure 2c). Further analyses suggest that our comparatively long sessions (40 min) may have reduced the influence of a systematic bias in our experiments (Supporting Text S1, Figure S1).

Considering the above findings, one reason why the winner of the previous intermittent presentation sequence often recurs during the current intermittent presentation sequence could be that it has enjoyed much dominance in the past. That is, if it is true that prior dominance in general increases the probability a percept will recur after a blank, then the extensive dominance during the previous intermittent presentation sequence (Figure 2a) could underlie the previous winner's elevated probability of regaining dominance during the current intermittent presentation sequence.

### The influence of longer continuous presentation episodes on stabilization

Would the winner of the previous intermittent presentation sequence still predominate during the current intermittent presentation sequence in case a more extensive period of spontaneous switching intervened, thereby moving the previous intermittent presentation sequence further into the past? Our second experiment was similar in design to the first one, but continuous presentation episodes were allowed to include up to four perceptual switches instead of just one. The delay between the final switch and the end of continuous presentation was no longer varied but fixed at the same duration as one intermittent presentation (∼0.5 s).

Figure 2d shows the subject-averaged probability that the current intermittent presentation sequence was won by the same percept as the previous intermittent presentation sequence, as a function of the number of intervening switches. Indeed, the probability decreased with an increasing number of switches, with almost no memory of the previous winner left after four perceptual switches. Figure 2e (blue curve) quantifies how this memory decay progressed over time, by depicting the same probability as a function of the duration of the intervening continuous presentation episode, for one representative subject. It reveals a gradual reduction with increasing duration, and the probability reaches chance level after a minute or so. This indicates that the influence of the previous winner fades during continuous viewing and is completely gone after about a minute.

In Figure 2e we again controlled for a potential role of a systematic preference for one of the two percepts, by reanalyzing the data separately for occasions where percept 1 won the previous intermittent presentation sequence (red curve) and those where percept 2 did (green curve). The red curve runs slightly above the green curve, indicating an overall tendency for this subject to perceive percept 1 more than percept 2. Nevertheless, the similarity between these curves confirms that our results do not depend on a systematic percept bias. Indeed, the systematic bias in this experiment was only 0.52 for this subject, and 0.56 on average.

### The influence of dominance during continuous presentation on stabilization

The above findings support the idea that dominance of a given percept in the recent past facilitates its regained dominance at stimulus reappearance. Consistent with this idea, the preference toward the previous winner decreased as a longer period of alternating dominance separated the moment of stimulus reappearance from the winner's dominance streak during the previous intermittent presentation sequence. A more specific prediction from the hypothesis, however, is that the preference toward the previous winner should not decay passively during continuous viewing. Instead, as was also suggested by our first experiment (Figure 2b), the evolution of the preference during continuous viewing should depend on what is being perceived. Hence, perception during the current intermittent presentation sequence should depend on the previous winner as well as on perception during the intervening period of continuous presentation.

**Figure 3:**
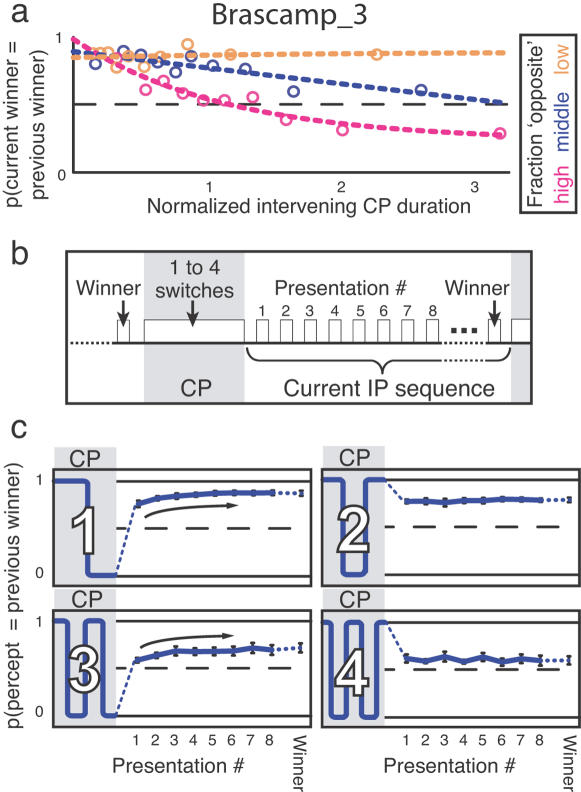
The influence of dominance throughout a continuous presentation episode on stabilization. a, The data of the second experiment were regrouped according to the *fraction of opposite dominance*; that is, the fraction of the time during a continuous viewing period that subjects experienced the percept opposite to the previous winner. The relation between the probability for the previous winner to also win the current intermittent presentation sequence (y-axis) and the intervening continuous presentation duration (x-axis) is affected by this fraction. The probability decays faster during continuous viewing periods containing mainly opposite dominance (magenta) than during periods containing more dominance of the previous winner itself (blue and orange). For this panel the durations of continuous presentation periods were normalized per subject by dividing by their mean continuous presentation duration (17 s on average), and then pooled over subjects. Data were split into three quantiles of the fraction of opposite dominance. Normalized continuous presentation durations for each of the three groups were then divided into ten quantiles to yield ten data points. The magenta and blue data points show a negative trend (p<0.01, Spearman; ρ = −0.98 and –0.68, respectively), but the orange ones do not (p>.25; ρ = −0.07). Note that, overall, the opposite percept took up more time during continuous viewing than did the previous winner percept (average fraction of opposite dominance is 0.61). b, The data of the second experiment were reanalyzed to study perception during the first eight presentations of an intermittent presentation sequence, as well as during the winning presentation. c, Probability that the previous winner dominates on individual presentations of the current intermittent presentation sequence, for up to four intervening perceptual switches (digits and diagrams on the left). In those conditions where continuous viewing ended in the percept opposite to the previous winner (after one or three switches), the probability of perceiving the previous winner is lower during the initial intermittent presentations following continuous viewing than during later presentations (trend marked by arrows; Spearman p<0.01; ρ = 0.88 for both one and three switches). Error bars indicate standard errors (n = 7).

Figure 3a depicts additional analyses of the second experiment that confirm this prediction and refine it. Like Figure 2e, Figure 3a shows the probability that the current intermittent presentation sequence is won by the winner of the previous intermittent presentation sequence, as a function of the intervening continuous presentation duration. To assess how perception during continuous viewing affects this probability, we now separated our data according to the fraction of the intervening continuous viewing period that was taken up by dominance of the percept opposite to the previous winner. This fraction could vary because spontaneous switches occur at random intervals during continuous viewing, and also because the number of switches varied. During continuous presentation periods where the opposite percept dominated a large fraction of the time (magenta), the bias toward the previous winner decayed rapidly over time, and eventually even turned into an opposite bias. If the continuous presentation period contained little opposite dominance, in contrast (orange), the bias toward the previous winner remained strong even after prolonged continuous viewing. The curve for an intermediate fraction of opposite dominance (blue) falls in between these two extremes. These results confirm that the preference for the previous winner does not decay passively during continuous viewing. Instead, it decays rapidly during dominance of the opposite percept but stays high during dominance of the previous winner percept itself. This is consistent with the notion that perception at stimulus reappearance reflects a balance that continuously evolves while viewing an ambiguous stimulus, ever shifting toward the currently dominant percept.

The final analysis goes beyond a comparison of the winners of consecutive intermittent presentation sequences, to include perception throughout an intermittent presentation sequence (Figure 3b). We now investigated perception during the first eight presentations of the current intermittent presentation sequence, as well as during the winning presentation (which could be either the eighth or a later one, depending on perception). Figure 3c shows the probability that the previous winner dominated on these nine presentations, with the number of intervening perceptual switches during continuous viewing depicted in diagrams on the left. The plots confirm that the tendency toward the previous winner decays with an increasing number of intervening switches. More importantly, however, they show an influence of the final percept during continuous viewing on perception during the subsequent intermittent presentation sequence. This is visible in the conditions where the intervening continuous presentation period contained either one or three perceptual switches (left plots), and therefore ended in the percept opposite to the previous winner (see diagrams). In those conditions, subjects reported the previous winner less often during the initial presentations of an intermittent presentation sequence than during later presentations. The resulting trend is marked by curved arrows. In the conditions involving two or four switches, in contrast, where the last percept during continuous viewing was identical to the previous winner, no such trend is visible.

An interesting aspect of the influence of the final percept of continuous viewing (Figure 3c) is its short duration in comparison to the minute-scale trace left by the winner of the previous intermittent presentation sequence (Figure 2e). The influence of the final percept of a continuous presentation period dissipates within a few intermittent presentations, or about 10 seconds. In our design, the winning percept dominated on eight consecutive intermittent presentations, whereas the final percept of a continuous viewing period dominated only briefly. This suggests that the longer a percept has dominated, the more persistent a bias it leaves. Moreover, it appears that a transient bias toward the most recent percept and a persistent bias toward the previous winner can exist simultaneously and independently, in the sense that the bias toward the previous winner is not erased by the transient bias toward the most recent percept. Instead, as soon as the transient bias wears off during the course of an intermittent presentation sequence, the bias toward the previous winner turns out to be unaffected (Figure 3b). This rebound toward the previous winner after a temporary tendency toward the opposite percept is even more pronounced if one externally forces perception to the opposite interpretation by means of a disambiguated stimulus, instead of waiting for a spontaneous switch to occur during continuous presentation (Supporting Text S1, Figure S3). These data suggest the existence of multiple parallel biases, each reflecting a different timescale of perceptual history.

### A model account based on multi-timescale adaptation

We interpret our findings as follows. *(i)* During perceptual dominance the visual system accumulates a bias toward the currently dominant percept. *(ii)* This accumulation takes place on several timescales, such that prolonged dominance (e.g. during intermittent presentation) leaves a persistent biasing trace, whereas brief dominance (e.g. just before the end of a continuous presentation period) leaves a more transient trace. *(iii)* Separate timescales work independently, such that the system can briefly be biased toward one percept without losing its longer-term bias toward the other percept. *(iv)* These biases become evident in perception when an ambiguous stimulus reappears after an interruption (rather than during ongoing viewing). Then, the visual system's choice between both percepts reflects the balance between various biasing traces that have so far accumulated. It is worth emphasizing that this approach treats perceptual stabilization as a repeated choice for the same percept on many stimulus onsets; not as persistence of a single perceptual state (during stimulus absence the system is in neither perceptual state). What is to be explained, therefore, is how perceptual history can make the system choose one percept over the other at stimulus onset.

We have constructed a computational model (Supporting Text S1, Figure S4) that implements the above four concepts. The model, an extension of [6], attributes perceptual stabilization to a history-driven bias in percept choice at stimulus onset. It is a natural property of the model that this bias takes effect specifically at stimulus appearance, and not during continuous viewing. An indication that this is an appropriate property is the experimental finding that factors that drive dominance at stimulus onset need not have a similar effect during prolonged viewing [10].

**Figure 4:**
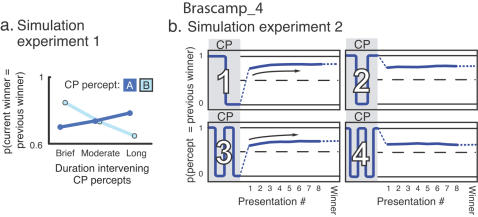
Model simulations. Our key findings are replicated by a model in which information on past perception is contained in the adaptation states of sensory neurons. a, Influence of one intervening perceptual switch on perceptual stabilization (cf. Figure 2b). The relation between percept durations A and B (see Figure 2a) and perceptual stabilization is replicated by the model. b, Influence of multiple intervening perceptual switches on perceptual stabilization (cf. Figure 3c). The probability that the previous winner is perceived during the current intermittent presentation sequence decreases with an increasing number of intervening switches, in line with the experimental data. In case the final percept during continuous viewing was opposite to the previous winner (one or three switches) the probability rises during the current intermittent presentation sequence (Spearman p<0.01; ρ = 0.95 and 0.92 for one and three switches, respectively), consistent with our experimental findings.

In the model the bias gradually accumulates during perceptual dominance, due to gradual sensitivity changes, or adaptation [11], [12], of neurons that code the currently dominant percept. The resulting imbalance in adaptation state persists for some time after the dominance period itself has ended, and therefore carries information on past perception–that is, acts as a memory store. At stimulus reappearance the difference in adaptation state between model neurons that code a recently dominant percept and those coding the other percept causes the recently dominant percept to win the competition. Interestingly, using only a single adaptation term with a single timescale of persistence, such as used by [6], we were unable to account for our findings. In contrast, when incorporating two adaptation terms, one with fast decay and the other more persistent, our data could be replicated in considerable detail (Figure 4).

## Discussion

Our results indicate that when a visual conflict reoccurs, traces of past perception increase the probability that the visual system will assume a previous state of interpretation rather than a different one. Despite its apparently simple nature, such a mechanism could have great merit for visual function. Visual input quite generally contains ambiguities, and in normal conditions only one perceptual interpretation is veridical. Selecting the one correct interpretation often requires the combination of multiple information sources [13] and engages extensive regions of the brain [14]. The current observations suggest that by biasing the system toward previous perceptual interpretations-be it the most recent one or one that consistently dominated longer ago-visual memory automatically enforces the outcome of previous perceptual conflicts, and thereby eliminates the need for the same conflicts to be resolved repeatedly.

Our model work shows that in neural terms this memory could be carried by the adaptation state of sensory neurons, provided adaptation occurs on more than one timescale. Indeed, adaptation in known to occur on a wide range of timescales in sensory cortex [11], [12], [15]–[18], and it has been argued on theoretical grounds that this holds promise for functional forms of history-dependence in neural systems [19], [20]. The present work thus suggests that the perceptual memory observed here constitutes one such functional correlate.

Our observations seem to conflict with an earlier study [9] where percept choice at stimulus reappearance was interpreted as an attempted continuation of the preceding percept sequence. For instance, a sequence of percepts ABA would cause percept B at reappearance, forming the regular sequence ABAB. This is inconsistent with our data mainly because it involves suppressive effects of past dominance on subsequent percept choice (for instance when ABA causes B whereas BBA causes A, the initial percept stimulates opposite perception at the end of the sequence), whereas we find only facilitation. The discrepancy may be due to the fact that the stimuli used by [9] were not fully ambiguous but biased toward one interpretation. The use of ambiguous stimuli throughout our experiments has allowed us to characterize memory of the internally constructed interpretation of a stimulus in isolation. Effects of unbalanced stimuli are probably a combination of the present effects and differential gain control at stages prior to where the percept arises [6]. This is known to cause different effects on subsequent perception [7], [21], [22].

We modeled our findings by adding a longer adaptation timescale to an existing model of perceptual stabilization [6]. An alternative model [23] of stabilization has also been proposed. Both models are essentially standard oscillator models expanded with an additional interaction [6] or storage mechanism [23] to allow a trace of previous perception to bias the next percept choice at stimulus reappearance. Both models in their original form have the limitation of lacking multiple timescales of storage. Regardless of the number of timescales, a drawback of [23] is that is predicts stabilization of a percept that has dominated briefly before stimulus offset but no stabilization of a percept that has dominated longer [Figure 7 in 23]. This is opposite to the experimental finding that brief dominance will prevent stabilization, and longer dominance is required for a percept to recur [Figure 2b in the present work, Figure 3c in 3]. Models of the type of [6] do reproduce this feature. Arguably a second objection to [23] is that it entails a binary memory, where the system is in one of two states of ‘remembering’ either percept. Experiments indicate that, instead, the system's bias toward one or the other percept varies over time in a continuous fashion [Figures 2b, 2e and 3a in the present work, also 24], consistent with the model we used.

Our view of ambiguous figure memory suggests a relation to visual memory in other situations. Previous notions that attributed perceptual stabilization to prolongation of a perceptual state during stimulus absence seemed to imply that it is a specifically ambiguity-related phenomenon. The present view of perceptual stabilization as a bias in a decision network–in this case regarding a perceptual decision at stimulus onset–allows more room for extensions beyond ambiguous perception. Specifically, the accumulation of a bias during perceptual dominance that we observe here is reminiscent of the progressive decrease in response time that is observed when subjects direct their attention [1], [25] or eye fixation [26], [27] to a similar search target appearing on several consecutive trials. This type of attention priming occurs automatically, independent of conscious recollection. It has been attributed to progressive use-related changes that build up in the neural structures activated when the target is attended [25], [27], so that every allocation of attention or gaze to an item simultaneously acts to stimulate reorientation to that item in the future. This is analogous to the accumulating bias that facilitates repeated perceptual dominance in our paradigm, a similarity that is particularly remarkable considering the numerous other parallels between attentional selection and perceptual dominance [14], [28].

Our findings bear directly on the question asked at the outset, how long a history to incorporate into current processing. Functionally, the answer depends on the liability for the conditions to change. If they change every few seconds it is useless to incorporate a minute-scale history because what happened a minute ago bears little relation to the present situation. If, in contrast, the conditions remain relatively stable for minutes, incorporating a longer-term history prevents unfavorable sensitivity to seconds-long (noisy) excursions. Our findings suggest how just such a strategy is implemented in vision, by use of parallel biasing traces on several timescales. In case of ambiguity resolution, if recent perception was highly stable, slow biases have built up sufficiently to outweigh the fast bias due to the most recent percept. If perception was variable, however, no slow biases have accumulated and the most recent percept becomes the main driving factor. This organization therefore ensures automatic adjustment of the effective memory timescale, dependent on the changeability of the situation at hand.

## Materials and Methods

### Subjects and task

Subjects were two authors and sixteen naive observers. All had normal or corrected-to-normal acuity. After showing subjects the stimuli and explaining their task, we orally obtained an informed consent statement before proceeding with the experiment. All experiments were conducted in agreement with Utrecht University ethics and safety guidelines. Three subjects showed a strong preference for one of the percepts during pilot experiments, and were not included in further testing. The remaining subjects had an average preference, as measured by the fraction of all intermittent presentation sequences won by their preferred percept, of 0.57 (σ = 0.05) and 0.61 (σ = 0.06) for the sphere and rivalry, respectively. Subjects were instructed to fixate the center of the display passively, and report their percepts via key presses. Experimental sessions took 40 minutes.

### Apparatus and stimuli

Ambiguous stimuli were an ambiguous rotating sphere (r = 0.65 deg; ω = 2.23 rad/s; 90 black dots of r = 0.02 deg; dot lifetime = 1 s) and dichoptic +/−45 deg grayscale Gabor patches (σ = 0.37 deg; 100% contrast; spf = 2.7 c/deg). Stimuli were presented on a gray background (35 cd/m^2^) within a white alignment ring (r = 1.7 deg) and with a red plus sign (side = 0.2 deg) marking fixation for the sphere. They were presented via a mirror stereoscope, on a CRT monitor (1600×1200 dpi) at a visual distance of 47 cm.

### Intermittent presentation sequences

The timing of intermittent presentation was optimized for each subject beforehand, to find a regime with robust perceptual stabilization. We therefore designed an adaptive procedure that dynamically adjusted stimulus timing according to a subject's perceptual reports, until no alternation was reported during 60 s of intermittent presentation. The average presentation duration was 0.5 s for both stimuli; the average blank duration was 1.4 s for binocular rivalry and 1.2 s for the ambiguous sphere. In all experiments we terminated and discarded an IP sequence if a subject did not reach a stable percept within 24 intermittent presentations. This happened on 1.3% of the occasions.

### Continuous viewing periods

The blank interval between the end of continuous viewing and the initial intermittent presentation was equal to the interval between consecutive presentations during intermittent viewing. In the first experiment the delay between the single perceptual switch and the end of continuous viewing was either 0.5, 1.5 or 3 s. A continuous viewing period was discarded if a second switch occurred during the delay, which for the three delay durations happened on 1, 13 and 29% of the occasions, respectively. In the second experiment the number of spontaneous switches varied randomly from 1 to 4 within sessions. The delay between the final switch and the end of continuous viewing was chosen equal to the duration of one presentation during intermittent viewing. This delay was chosen for experimental efficiency, because by design a presentation duration during intermittent viewing was short enough to minimize the occurrence of additional switches before stimulus offset. The analysis of memory decay during continuous presentation (Figures 2e, 3a and S2) required more data than the other analyses, and was based on additional sessions with three naive observers of the sphere.

### Forced perceptual switches

In the experiments where perception was exogenously forced away from the previous winner (Figure S3), unambiguous stimuli were constructed as follows. For the ambiguous sphere we added binocular disparity to the dots, defining a unique rotation direction. Brief exposure to such an unambiguous rotation direction tends to cause perception of that *same* rotation direction during subsequent ambiguous viewing [29]. For binocular rivalry the unambiguous stimulus consisted of one of the eyes' images in isolation, which caused dominance of the *opposite* eye's image during subsequent ambiguous viewing (flash suppression [30]). An effective duration of unambiguous presentation was determined per subject in pilot sessions beforehand, and amounted to 0.9 s on average for the sphere, and 0.8 s on average for binocular rivalry.

## Supporting Information

Supporting Figure S1 (1.11 MB EPS)

Supporting Figure S2 (0.82 MB EPS)

Supporting Figure S3 (0.75 MB EPS)

Supporting Figure S4 (1.00 MB EPS)

Text S1 Supporting text
(0.05 MB DOC)

## References

[1] Maljkovic V, Nakayama K (2000). Priming of popout III: A short-term implicit memory system beneficial for rapid target selection.. Visual Cognition.

[2] Ernst MO, Banks MS, Bulthoff HH (2000). Touch can change visual slant perception.. Nat Neurosci.

[3] Leopold DA, Wilke M, Maier A, Logothetis NK (2002). Stable perception of visually ambiguous patterns.. Nat Neurosci.

[4] Maier A, Wilke M, Logothetis NK, Leopold DA (2003). Perception of temporally interleaved ambiguous patterns.. Curr Biol.

[5] Orbach J, Ehrlich D, Heath HA (1963). Reversibility of the Necker Cube. I. An Examination of the Concept of “Satiation of Orientation”.. Percept Mot Skills.

[6] Noest AJ, Van Ee R, Nijs MM, Van Wezel RJA (2007). Percept choice sequences driven by interrupted ambiguous stimuli: a low-level neural model.. J Vis.

[7] Pearson J, Clifford CW (2005). Mechanisms selectively engaged in rivalry: normal vision habituates, rivalrous vision primes.. Vision Res.

[8] Chen X, He S (2004). Local factors determine the stabilization of monocular ambiguous and binocular rivalry stimuli.. Curr Biol.

[9] Maloney LT, Dal Martello MF, Sahm C, Spillmann L (2005). Past trials influence perception of ambiguous motion quartets through pattern completion.. Proc Natl Acad Sci U S A.

[10] Carter O, Cavanagh P (2007). Onset rivalry: brief presentation isolates an early independent phase of perceptual competition.. PLoS ONE.

[11] Ohzawa I, Sclar G, Freeman RD (1985). Contrast gain control in the cat's visual system.. J Neurophysiol.

[12] Bonds AB (1991). Temporal dynamics of contrast gain in single cells of the cat striate cortex.. Vis Neurosci.

[13] Ernst MO, Bulthoff HH (2004). Merging the senses into a robust percept.. Trends Cogn Sci.

[14] Leopold DA, Logothetis NK (1999). Multistable phenomena: changing views in perception.. Trends Cogn Sci.

[15] Muller JR, Metha AB, Krauskopf J, Lennie P (1999). Rapid adaptation in visual cortex to the structure of images.. Science.

[16] Ulanovsky N, Las L, Farkas D, Nelken I (2004). Multiple time scales of adaptation in auditory cortex neurons.. J Neurosci.

[17] Albrecht DG, Farrar SB, Hamilton DB (1984). Spatial contrast adaptation characteristics of neurones recorded in the cat's visual cortex.. J Physiol.

[18] Rose D, Lowe I (1982). Dynamics of adaptation to contrast.. Perception.

[19] Drew PJ, Abbott LF (2006). Models and properties of power-law adaptation in neural systems.. J Neurophysiol.

[20] Gilboa G, Chen R, Brenner N (2005). History-dependent multiple-time-scale dynamics in a single-neuron model.. J Neurosci.

[21] Brascamp JW, Knapen THJ, Kanai R, Van Ee R, Van den Berg AV (2007). Flash suppression and flash facilitation in binocular rivalry.. J Vis.

[22] Kanai R, Verstraten FA (2005). Perceptual manifestations of fast neural plasticity: motion priming, rapid motion aftereffect and perceptual sensitization.. Vision Res.

[23] Wilson HR (2007). Minimal physiological conditions for binocular rivalry and rivalry memory.. Vision Res.

[24] Pastukhov A, Braun J (2007). Temporal characteristics of priming effects on the perception of ambiguous patterns [Abstract].. J Vis.

[25] Kristjansson A (2006). Rapid learning in attention shifts: A review.. Visual Cognition.

[26] McPeek RM, Maljkovic V, Nakayama K (1999). Saccades require focal attention and are facilitated by a short-term memory system.. Vision Res.

[27] Dorris MC, Pare M, Munoz DP (2000). Immediate neural plasticity shapes motor performance.. J Neurosci.

[28] Mitchell JF, Stoner GR, Reynolds JH (2004). Object-based attention determines dominance in binocular rivalry.. Nature.

[29] Jiang Y, Pantle AJ, Mark LS (1998). Visual inertia of rotating 3-D objects.. Percept Psychophys.

[30] Wolfe JM (1984). Reversing ocular dominance and suppression in a single flash.. Vision Res.

