# Supplementary material for: Multi-Timescale Perceptual History Resolves Visual Ambiguity
Source: PLoS One. 2008 Jan 30;3(1):e1497. doi: 10.1371/journal.pone.0001497 (PMC2204053; doi:10.1371/journal.pone.0001497)
Supplement: Text S1 — Supporting text (0.05 MB DOC) [file pone.0001497.s005.doc]

# **Supporting Information**

### Perceptual stabilization and bias in binocular rivalry

Figure S1a shows the probability for binocular rivalry of perceiving the winner of the previous intermittent presentation sequence during the current intermittent presentation sequence, for up to four intervening perceptual switches during continuous presentation. The results are similar to those for the ambiguous sphere. The probability lies above chance level throughout all four plots, and the conditions where the last percept preceding the current intermittent presentation sequence was opposite to the previous winner (one and three switches) reveal an influence of this final percept. In those conditions, the probability of perceiving the previous winner was lower during the initial intermittent presentations than during the later ones (rise indicated by arrows). One qualitative difference with the ambiguous sphere data is that for binocular rivalry the influence of the final percept of the continuous viewing episode seems stronger. Part of its effect extends to the winner of an intermittent presentation sequence. As a consequence, the probability for the current winner to equal the previous winner does not decay monotonically with the number of intervening switches (as it did for the ambiguous sphere), but lies slightly higher after even switch numbers than after odd ones.

Like those of the ambiguous sphere, our observers of binocular rivalry displayed only a modest systematic perceptual bias (the fraction of intermittent presentation sequences won by the preferred percept was 0.61 on average). This seems to conflict with the finding [1] that systematic bias can profoundly influence binocular rivalry perception during intermittent presentation. We tested if our comparatively long session duration (40 min, versus 2-10 min in [1]) could explain this discrepancy. Figure S1b shows the time course of one typical session, plotting the winners of consecutive intermittent presentation sequences against time. Both percepts have periods (runs) during which they won several consecutive intermittent presentation sequences (the circled numbers indicate the number of consecutive intermittent presentation sequences). Of note, the typical duration of such a run is several minutes. Now suppose that one effect of a systematic bias is that the first percept to get stabilized within a session is often the same for a given subject. Because runs of repeated dominance typically take several minutes, such a scenario would cause a profound bias in case the session duration is of the order of minutes, whereas longer sessions would reduce the influence of bias. In Figure S1c we investigate this idea by calculating the bias of our binocular rivalry observers for consecutive five-minute blocks within sessions. Indeed, the bias during the first block is considerably larger than the overall bias, in support of the idea that our use of long sessions may have reduced the influence of a consistent bias.

*The influence of a forced perceptual switch*

We performed an additional experiment where we externally forced perception away from the previous winner, instead of waiting for spontaneous switches to occur during continuous presentation. We interleaved intermittent presentation sequences, not with continuous viewing episodes, but with presentation of an unambiguous stimulus (UP; Figure S3a) that imposed the percept opposite to the previous winner. For binocular rivalry we used *flash suppression* [2]; for the ambiguous sphere we used positive priming with a disparity-disambiguated sphere [cf. 3]. Figure S3b depicts how this affected perception during intermittent presentation. During the initial presentation following unambiguous stimulation the probability of perceiving the previous winner was relatively low, indicating successful disruption of the winner’s dominance streak. However, during the following intermittent presentations the probability of perceiving the previous winner gradually recovered. This pattern of results is similar to that observed when spontaneous switches ended in the percept opposite to the previous winner (Figures 3c and S1a), and demonstrates that the influence of the previous winner can survive both spontaneous and forced perceptual switches.

# A model based on multi-timescale adaptation

Recurrence of the most recent percept after a blank has previously been accounted for using a modified version of the standard bistable oscillator model [4]. In such models, the two perceptual interpretations correspond to neural populations that interact via mutual inhibition and that exhibit slow self-adaptation. In the model by [4] past perception influences percept choice at stimulus reappearance via the adaptation component, which persists for some time after the model input (stimulus) has been switched off, and after the populations’ responses themselves have as a result fallen to near baseline.

Each population, indexed *i* and *j*, is modeled by two differential equations; one for its neural response *H* and one for its adaptation state *A*. We show the equations for *i*; those for *j* are the same with indices *i* and *j* exchanged. The response *H_i_* is given by

(1)

*X_i_* is *i*’s excitatory input, *γ* is a constant that determines the strength of cross inhibition and *S*[*H_j_*] is a sigmoid function of *H_j_* (*H* itself can be viewed as a population-averaged membrane potential, and *S*[*H*] as a population-averaged firing rate). The term -*H_i_*(1+*A_i_*), here called the ‘shunting’ term, implements a standard diminishing effect of adaptation *A* on activity *H*, causing switches during continuous viewing. The term +*βA_i_*, here called the ‘additive’ term, is a key component in [4] that mediates a facilitatory effect of adaptation *A* at stimulus onset (*β* is a constant). Adaptation is implemented as ‘leaky integration’ over activity *S*[*H_i_*]:

(2)

Here *τ*>1 is a time constant and *α* is a constant.

When both activities *H_i_* and *H_j_* start rising from near baseline after a blank period (during which *X_i_*= *X_j_*=0) their race is biased by adaptations *A_i_* and *A_j_*. Here the ‘shunting’ term acts against the more adapted percept and the ‘additive’ term acts in its favor. Figure S4a illustrates how the outcome of the competition between these forces depends on the specific combination of *A_i_* and *A_j_* at stimulus onset. The gray and white regions indicate combinations of *A_i_* and *A_j_* that lead to onset dominance of percept *i* and *j*, respectively. Crucially, the model predicts a region, below the dotted line, where the ‘additive’ component prevails and the more adapted percept gains dominance. In earlier models, that did not incorporate the ‘additive’ term, this region was absent. The presence of this region allows the model to produce perceptual stabilization, where the more adapted percept keeps on regaining dominance on consecutive presentations.

With a single time scale of adaptation (eq. 2) this model can thus explain recurrence after a blank of the most recent percept. The present findings, however, require the inclusion of adaptation on multiple timescales in the model. The most compelling reason for this is the fact that the winner of the previous intermittent presentation sequence recurs during the following intermittent presentation sequence regardless of what the final intervening percept was. This cannot be explained by the model in its present form. In the following we will explain the reason for this limitation, and we will show how it is remedied by multiple adaptation timescales.

The course of adaptations *A_i_* and *A_j_* during perceptual alternations in the existing model is plotted in Figure S4b, both as a function of time (left) and in the (*A_i_*, *A_j_*)-space (right) introduced in panel a. Importantly, in the right plot the course of adaptation during dominance of *i* (from I to II) is the same as its course during dominance of *j* (from II back to I), but mirrored in the main diagonal. Note that the distribution of gray in Figure S3a is also identical to the distribution of white mirrored in the main diagonal. These two facts combined imply the following: Whenever a blank period introduced during dominance of *i* has characteristics that lead to recurrence of *i*, the model in its present form predicts that an identical blank period introduced during dominance of *j* will lead to recurrence of *j*. This is illustrated in panels c and d. Panel c shows how a blank period introduced during dominance of *j* can lead to recurrence of percept *j*. Both the delay period between the switch to *j* and stimulus offset (II to III), and the blank duration (III to IV) are sufficiently long for the adaptation states to end up in a white region at stimulus reappearance (IV), causing *j* to regain dominance. Panel d illustrates the effect of a blank period with identical timing, but introduced during dominance of *i*. The resulting course of adaptation in the (*A_i_*, *A_j_*)-space is the mirror image of that in panel c, and because the gray and white regions are also each other’s mirror image, percept *i* recurs. In order to explain our present finding that the previous winner tends to recur at stimulus reappearance, regardless of whether the stimulus was removed during dominance of *i* or during dominance of *j*, we must break the symmetry of either the adaptation trajectories, or the underlying gray-and-white landscape. The latter is what the introduction of a second adaptation time scale does.

We expand the model with a second, slower, adaptation term:

(3)

(4)

(5)

These equations are identical to (1) and (2), except that where previously there was only one *A* there now are two. Subscripts and superscripts *S* and *F* indicate slow and fast adaptation, respectively. Consequently, *τ_S_*>*τ_F_*.

Figure S4e shows the course of both fast and slow adaptations during an intermittent presentation sequence and the following continuous presentation period. Slow adaptation (top graph) gradually accumulates during intermittent presentation, and stays asymmetrical throughout continuous viewing. In other words, it keeps on carrying a trace of the most recent winner, *i*, throughout continuous viewing, even as perception itself oscillates between *i* and *j*. Fast adaptation (bottom graph) is also asymmetrical during intermittent presentation, but *A_i_^F^* and *A_j_^F^* quickly resume their symmetrical oscillation cycles during continuous viewing . Fast adaptation during and right after the continuous presentation period is magnified in panel f. The stimulus is removed during dominance of *j*, and the course of fast adaptation is very similar to that in panel c. However, the outcome of the race between both percepts at stimulus reappearance (IV) is now determined by a combination of both fast and slow adaptation. Given the remaining asymmetry between slow adaptations *A_i_^S^* and *A_j_^S^*, the gray-and-white landscape as a function of *A_i_^F^* and *A_j_^F^* is no longer symmetrical. The gray region, which leads to dominance of the previous winner *i*, has expanded. As a consequence, *i* regains dominance after the blank, even though the final percept before the blank was *j*.

The settings during the simulations of Figure 4 were *γ*=3.3, *β_F_*=0.28, *β_S_*=0.45, *α_F_*=4.0, *α _S_*=0.4, *τ_F_*=90, *τ_S_*=800, *X*=1.0, all equal for *i* and *j*. For the sigmoid nonlinearity *S*[*H*] we used . The presentation time and blank time during intermittent presentation were 50 and 90, respectively (arbitrary units). In the simulation of Figure 4a the delay between a switch and stimulus offset was varied between 50, 75 and 100; during the simulation of Figure 4b it was always 50. For our simulations we used a fourth order Runge-Kutta method with step size 0.1, applying Poisson noise to *S*[*H*] on every time step. This was implemented as

*S*[*H*]_old_ are *S*[*H*]_new_ are the values before and after the addition of noise, respectively. *Poisson*(*μ*) is a random value from the Poisson distribution with mean *μ*, and *C* is a constant that we set to 120. The data in Figure 4 was generated using independent noise on *S*[*H*] in all six equations. We obtained equivalent results when applying identical noise to all three occurrences of *S*[*H_i_*], and to all three occurrences of *S*[*H_j_*].
